# Transcriptome analysis revealed differences in gene expression in sheep muscle tissue at different developmental stages

**DOI:** 10.1186/s12863-024-01235-9

**Published:** 2024-06-07

**Authors:** Sailuo Wan, Mengyu Lou, Sihuan Zhang, Shuang Li, Yinghui Ling

**Affiliations:** 1 College of Agricultural Engineering, Anhui Open University, Hefei, 230022 P.R. China; 2 College of Animal Science and Technology, Anhui Agricultural University, Hefei, 230036 P.R. China; 3 Anhui Province Key Laboratory of Local Livestock and Poultry Genetic Resource Conservation and Bio-Breeding, Anhui Agricultural University, Hefei, 230036 P.R. China

**Keywords:** Sheep, Skeletal muscle, Muscle development, Transcriptome sequencing

## Abstract

**Background:**

The analysis of differentially expressed genes in muscle tissues of sheep at different ages is helpful to analyze the gene expression trends during muscle development. In this study, the longissimus dorsi muscle of pure breeding Hu sheep (H), Suffolk sheep and Hu sheep hybrid F1 generation (SH) and East Friesian and Hu sheep hybrid sheep (EHH) three strains of sheep born 2 days (B2) and 8 months (M8) was used as the research object, and transcriptome sequencing technology was used to identify the differentially expressed genes of sheep longissimus dorsi muscle in these two stages. Subsequently, GO and KEGG enrichment analysis were performed on the differential genes. Nine differentially expressed genes were randomly selected and their expression levels were verified by qRT-PCR.

**Results:**

The results showed that 842, 1301 and 1137 differentially expressed genes were identified in H group, SH group and EHH group, respectively. Among them, 191 differential genes were enriched in these three strains, including pre-folding protein subunit 6 (*PFDN6*), DnaJ heat shock protein family member A4 (*DNAJA4*), myosin heavy chain 8 (*MYH8*) and so on. GO and KEGG enrichment analysis was performed on 191 differentially expressed genes shared by the three strains to determine common biological pathways. The results showed that the differentially expressed genes were significantly enriched in ribosomes, unfolded protein binding, FoxO signaling pathway, glycolysis / glycogen generation and glutathione signaling pathway that regulate muscle protein synthesis and energy metabolism. The results of qRT-PCR were consistent with transcriptome sequencing, which proved that the sequencing results were reliable.

**Conclusions:**

Overall, this study revealed the important genes and signaling pathways related to sheep skeletal muscle development, and the result laid a foundation for further understanding the mechanism of sheep skeletal muscle development.

**Supplementary Information:**

The online version contains supplementary material available at 10.1186/s12863-024-01235-9.

## Background

Skeletal muscle growth and development is an important factor affecting the yield and quality of mutton, which is finely regulated by many genetic and nutritional factors, and under a complex regulatory network, it undergoes multiple stages of proliferation, differentiation and fusion, and eventually becomes a mature muscle fiber [1, 2]. Muscle development is mainly divided into two stages: increase in the number of myoblasts, myotube formation and myofiber hypertrophy (increased protein levels). The role of hyperplasia on muscle growth is limited to the embryonic or a short period after birth, and postnatal muscle development is mainly dependent on myofiber hypertrophy [3, 4]. MRFs (Muscle regulatory factors) is essential in early muscle development. Among them, *Myf4* determines myogenic cell fate, *Myf5* is essential for myoblast proliferation, Myogenin regulates myoblast differentiation. *Myf5*, Myogenin and *MyoD* jointly guide myoblast fusion to form multinucleated muscle fibers [5]. Skeletal muscle hypertrophy is regulated by a variety of factors, including hormones, growth factors, signaling pathways and mechanical signals [6]. Transcription factors such as MEF2, SRF, PGC-1α4 and YAP promote myofiber growth. The FOXO family regulates the expression of catabolism-related genes. PI3K-AKT stimulates protein synthesis through activation of mTOR, a key pathway affecting muscle mass and metabolism [7].

In recent years, RNA-Seq has been used to explore the mechanisms of skeletal muscle growth and development. Studies have shown that the diameter of myofibers reached its maximum at 150 days and identified six genes involved in the development of skeletal muscle fibers, including *MSTN* (Myostatin) and *MYF6* (myogenic factor 6), through the analysis of the whole transcript sequence of the longissimus dorsi muscle of Ningxiang pigs at four different stages of postnatal life (Yu et al., 2023) [8]. It has been shown that using RNA-Seq to analyze the expression profiles of lncRNAs in three developmental stages of skeletal muscle in New Zealand rabbits and identified lncRNAs such as LINC-8613 and LINC-8705, which are involved in the proliferation and energy metabolism of skeletal muscle cells [9]. The whole transcriptome RNA-Seq analysis of goat dorsal muscles at four stages of prenatal and postnatal development have revealed that biological pathways such as Rap1 signaling pathway, PI3K-Akt signaling pathway, and AMPK signaling pathway are directly related to temporal changes of skeletal muscle development in goats [10]. Studies have shown that a series of genes related to muscle development, such as *MYL9* (myogenic factor 6) and *PAK1* (p21 activated kinase 1), were found in the skeletal muscle transcriptome sequencing results of Peking duck and Hanzhong duck at embryonic stage 17,21,27, and 6 months after birth [11].

However, there are few studies on the development of skeletal muscle in sheep at different stages. In this study, we analyzed the differentially expressed genes and signaling pathways in the longissimus dorsi muscle of three different populations of sheep at 2 days of age and 8 months of age by using transcriptome sequencing technology, with the aim of providing a reference to investigate the developmental mechanism of skeletal muscle in sheep.

## Materials and methods

### Preparation of experimental animals and collection of samples

This study was conducted with Hu sheep (H), F1 generation of Suffolk x Hu sheep hybrids (SH), and progeny from crossbreeding East Friesian and Hu sheep and then backcrossing with Hu sheep (EHH). Sheep used in the experiment were provided by Lujiang Xiangrui Breeding Co., Ltd. (Hefei, China). All sheep were reared under the same management conditions. Two 2-day-old sheep and three 8-month-old sheep of the three strains were randomly selected. The sheep were euthanized following the intravenous injection of a barbiturate (30 mg/kg), and their longissimus dorsi muscles were collected as experimental samples. Samples were rinsed three times with phosphate buffer (Dulbecco’s Phosphate-Buffered Saline, DPBS) containing 1× penicillin and streptomycin. All samples were placed in freezing tubes and snap-frozen immediately in liquid nitrogen and then stored at -80 °C.

### RNA extraction, library construction, and sequencing

Skeletal muscle was ground to a powder under liquid nitrogen and total RNA was isolated using Trizol reagent (Adderall Biologicals, Beijing, China). RNA concentration and purity were estimated using a Nanodrop one Micro UV-visible Spectrophotometer (Thermo Fisher Scientific, USA). RNA integrity and total amount were determined by the Agilent 2100 bioanalyzer bioassay. The extracted RNA was stored at -80 °C for further experiments. 5 µg of RNA from each sample was used as raw material for library construction. The poly-A mRNA was isolated using Oligo (dT) magnetic beads. Double-stranded cDNAs were synthesized from dNTPs, and the purified cDNAs were subjected to end repair and addition of polyA junctions, respectively. The cDNAs around 370 ~ 420 bp were screened for PCR amplification to construct a cDNA library. RNA samples were sequenced on the Illumina Hiseq 2000 platform after quality control of the library. The library construction and sequencing were done by Beijing Novogene.

### Quality control and transcript assembly

The raw data were filtered using fastp (version 0.19.7) software as follows: adaptor reads; unknown base information reads; and reads for which the number of bases with Qphred ≤ 20 accounted for more than 50% of the entire read length. Finally, Q20, Q30 and GC content of the clean data were calculated. The reference genome and gene annotation file (GCF_016772045.1) for sheep (*Ovis aries*) was downloaded from NCBI. Indexes of reference genomes were constructed using HISAT2 (v2.0.5) and compared. The transcripts were then assembled using StringTie (1.3.3b).

### Mining and analysis of the sequence data

FeatureCounts (1.5.0-p3) was used to calculate the expression level of a gene, expressed as FPKM (Fragments Per Kilobase of exon model per Million mapped fragments). DESeq2 (1.20.0) was used to analyzed for differences. *P*-value < 0.05 and |log_2_ Fold Change| ≥ 2 were set to screen for differentially expressed genes (DEGs).

### GO classification and KEGG enrichment analysis of differentially expressed genes

clusterProfiler (3.8.1) software was used to perform GO (http://www.geneontology.org) functional enrichment analysis and KEGG (http://www.kegg.jp) pathway enrichment analysis on the differential gene sets. *P*-value < 0.05 was set as significant enrichment, and *P*-value < 0.01 was set as extremely significant enrichment.

### GSEA enrichment analysis

GSEA (Gene Set Enrichment Analysis) analysis tool (http://www.broadinstitute.org/gsea/index.jsp) was used to perform GSEA analysis on GO and KEGG datasets of 2-day-old and 8-month-old sheep, respectively. *P*-value < 0.05 was set as the threshold for significant enrichment.

### RNA reverse transcription to cDNA

The reaction system was prepared according to the instructions of the reverse transcription kit RTIII All-in-One Mix with ds DNase (Monad Biotech Co., Ltd., Beijing, China). Ds DNase, 5 × RT III All-in-one Mix, template RNA and Nuclease-Free water were mixed on the ice box, gently blown and mixed, and then instantaneously separated. The reaction was incubated at 37 °C for 2 min to remove genomic DNA contamination, then incubated at 55 °C for 15 min, and then reaction incubated at 85 °C for 5 min to terminate the reaction. Synthesized cDNA was stored at -20 °C for subsequent real-time quantitative PCR (qRT-PCR).

### Real-time fluorescence quantitative PCR validation

Randomly selected 9 differentially expressed genes. Primer pairs were designed using NCBI Primer-BLAST and synthesized by TsingKe Biotechnology (TsingKe, Nanjing, China). The *GAPDH* internal reference gene was used as a control (Table 1). qRT-PCR was performed using a 2 × Q3 SYBR qPCR Premix (TOLOBIO, Shanghai, China) and CFX Connect Real-Time PCR Detection System (Bio-Rad, USA). qRT-PCR reactions were conducted in a final volume of 10 µL, comprising the following: 5 µL of 2 × Q3 SYBR qPCR Mix (High ROX), 4.1 µL of sterilized water, 0.5 µL of DNA template (cDNA solution), 0.2 µL of qRT-PCR forward primer (10 µmol/L) and 0.2 µL of qRT-PCR reverse primer (10 µmol/L). Amplification conditions were as follows: pre denaturation at 95 °C for 30 s followed by 40 cycles of 95 °C for 10 s and 60 °C for 30 s. Melting curve analysis was conducted at 95 °C for 15 s, 60 °C for 34 s, and 95 °C for 15 s.

**Table 1:** Primer sequences

Gene	Forward primer(5’~3’)	Reverse primer(5’~3’)	Product length/bp
GAPDH	CCACGCCATCACTGCCACCC	CAGCCTTGGCAGCGCCAGTA	249
CDKN1A	CCCAGGAGAGCCACAGGT	TCGAAGTTCCATCGCTCTCG	176
PCK2	CGGCTGAACACAAAGGGAAG	CCGGAACCAGTTGACATGGA	155
PFDN6	GAGGAGTTCGGGGTACTGC	CAGGGCCAGTTCCTCCTTC	199
MYH8	CCATCTACAAGCTCACGGGG	CAGCCTTGTCAGCCACTTCA	111
ACTC1	CTCACGGATTACCTCA	AGCAACATAGCACAGC	108
HYPK	GGCCATGTCCGTGATTGGAG	GCCCATGTGTTCCCTCAAGC	169
CCDC80	GCATGCAATTTTGGTCTGCG	GGTACGTCTTCTCGCTCCAC	125
IGF2	AGCTCGGCCATTCAGACATC	CTGGATGGTCGGCTGAAGTAG	191
MRPL41	AGCTCAAGCCCTACGTGAAC	CGTACCGTTCTAGGCGCTC	147

## Results

### RNA-Seq sequencing data showed good repeatability

After sequencing, an average of 44.09 million raw reads was obtained for 2-day-old sheep and 44.52 million raw reads for 8-month-old sheep, and screening yielded 44.52 million and 41.9 million reads, respectively. Among them, the sequencing comparison rates were 95.81% and 95.97% for 2-day-old and 8-month-old sheep, respectively. The sequencing error rates of the data were all below 0.03%, Q20 > 96.54%, Q30 > 90.61%, indicating valid sequencing data.

**Fig. 1:**
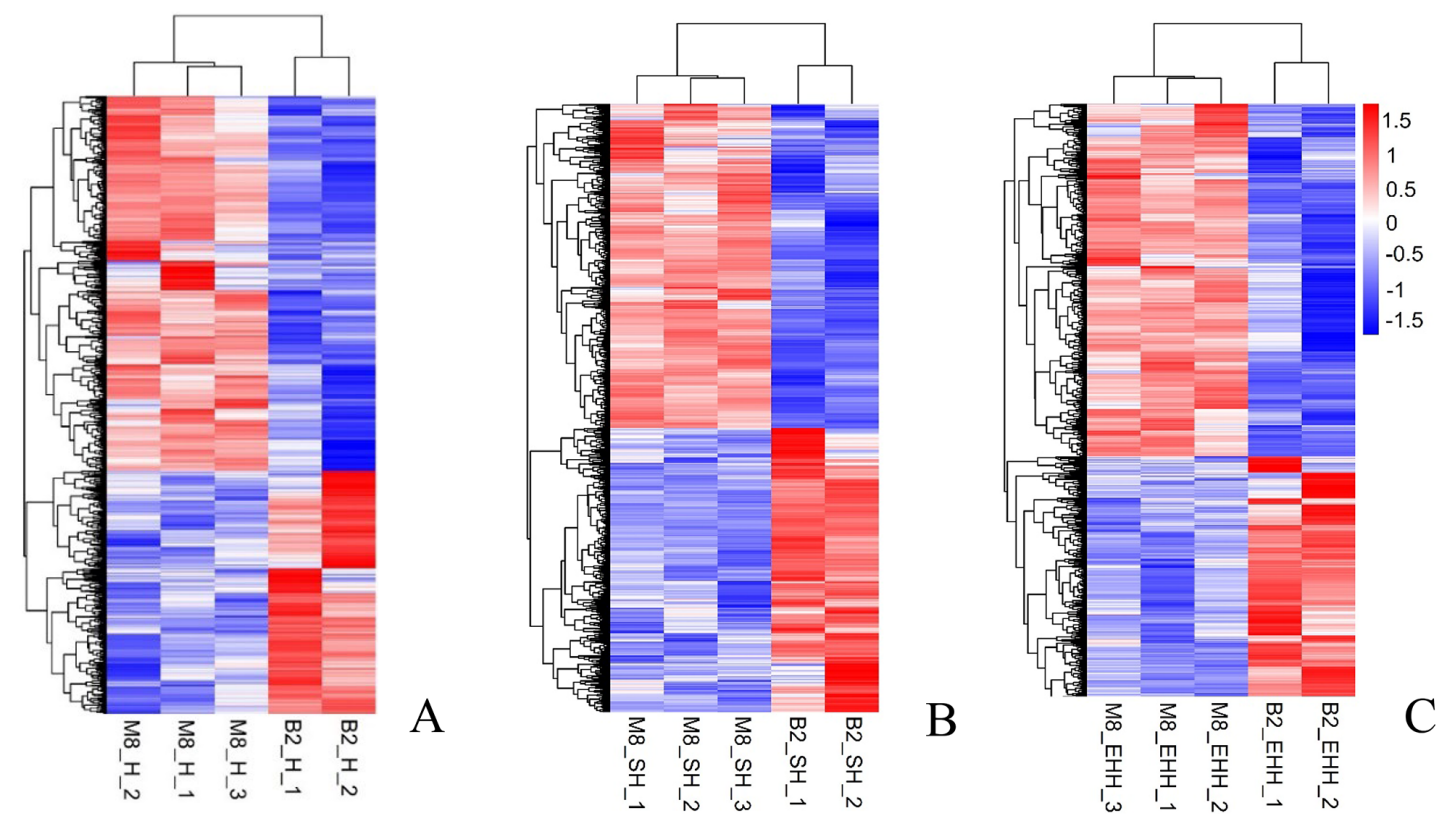
Heat map of differential gene clustering. B2, Born 2 days old; M8, 8 months; A. Pure breeding Hu sheep (H) group. B. Suffolk and Hu sheep crossbreed (SH) group. C. East Friesian and Hu sheep after hybridization backcross (EHH) group, same below

Cluster plots for the three strains show reproducibility of the samples, it shows that the transcriptome of 2-day-old and 8-month-old sheep is different (Fig. 1).

### DEGs at different stages are important in muscle development

**Fig. 2:**
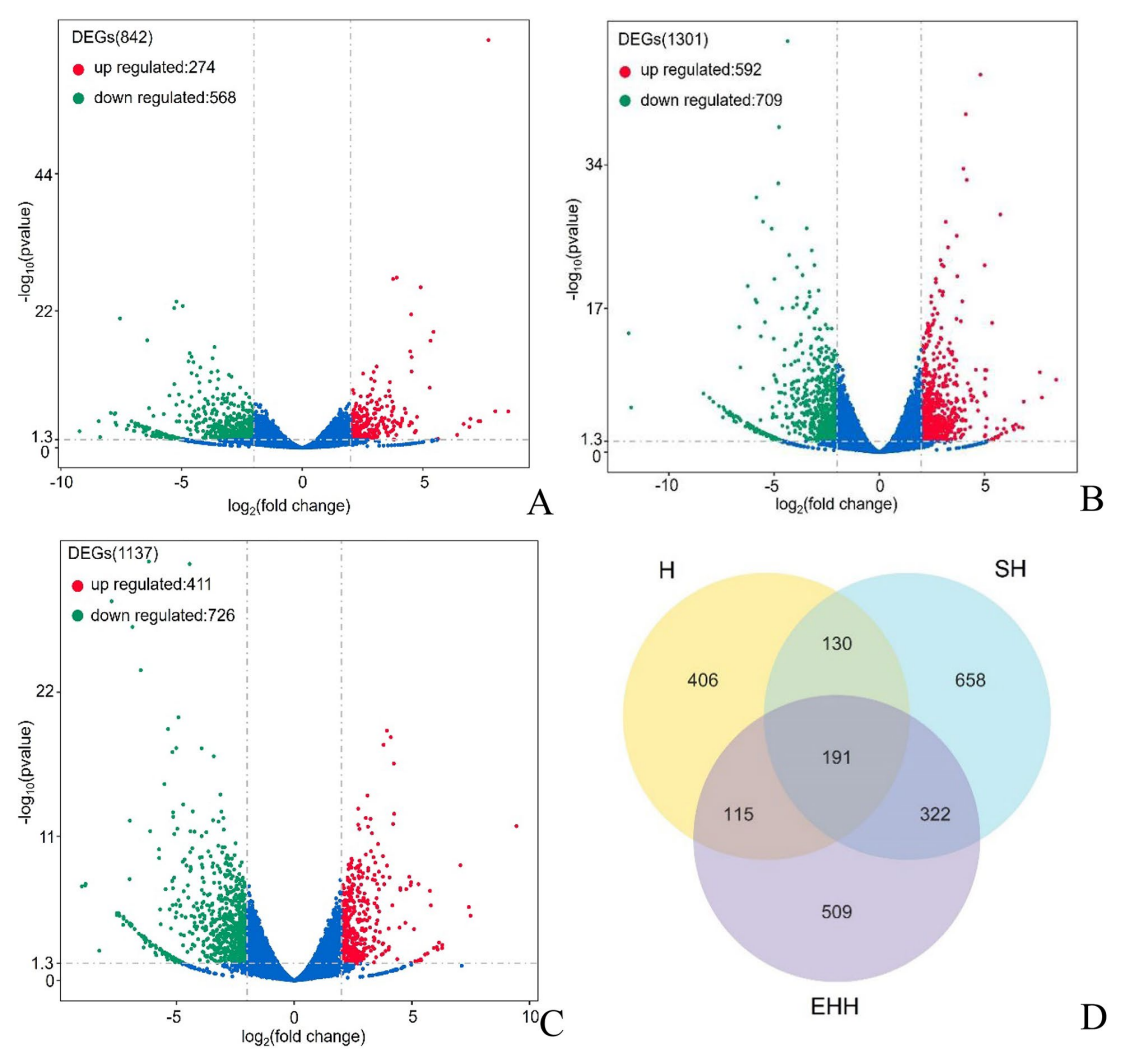
Differentially expressed gene analysis. A. Volcano map of differentially expressed genes in group H. B. Volcano map of differentially expressed genes in group SH. C. Volcano map of differentially expressed genes in group EHH. D. Venn diagram

842, 1301 and 1137 DEGs were identified in the B2 and M8 groups of the three strains, respectively, with 274 DEGs up-regulated and 586 DEGs down-regulated in the B2 compared to the M8 in H (Fig. 2A), 592 DEGs up-regulated and 709 DEGs down-regulated in the B2 compared to the M8 in SH (Fig. 2B), and 411 DEGs up-regulated and 726 DEGs down-regulated in the B2 compared to the M8 in EHH (Fig. 2C). The Venn plot identified 191 differential genes that were differentially expressed in all three strains (Fig. 2D), including *PFDN6*, *MYH8*, *DIAPH3*(diaphanous related formin 3),*CDKN1A* (cyclin dependent kinase inhibitor 1 A), *ALDH2* (aldehyde dehydrogenase 2 family member) and *BPGM* (bisphosphoglycerate mutase), which are related to skeletal muscle growth and development. *PFDN6* and *MYH8* have been found to play a role in new actin and microtubule chains, promoting protein synthesis and stabilizing actin cytoskeleton in the infancy [12, 13]. *DIAPH3* is a scaffold protein with the function of nucleation and extension of actin filaments [14]. A recent study showed that *CDKN1A* promotes skeletal muscle cell proliferation as a target gene of MiR-208b [15]. *ALDH2* and *BPGM* are involved in the glycolysis process [16, 17]. *CHAC1* is significantly expressed in skeletal muscle [18]. Suggesting these genes are involved in the regulation of skeletal muscle development in sheep.

### GO and KEGG enrichment analysis of differentially expressed genes

**Fig. 3:**
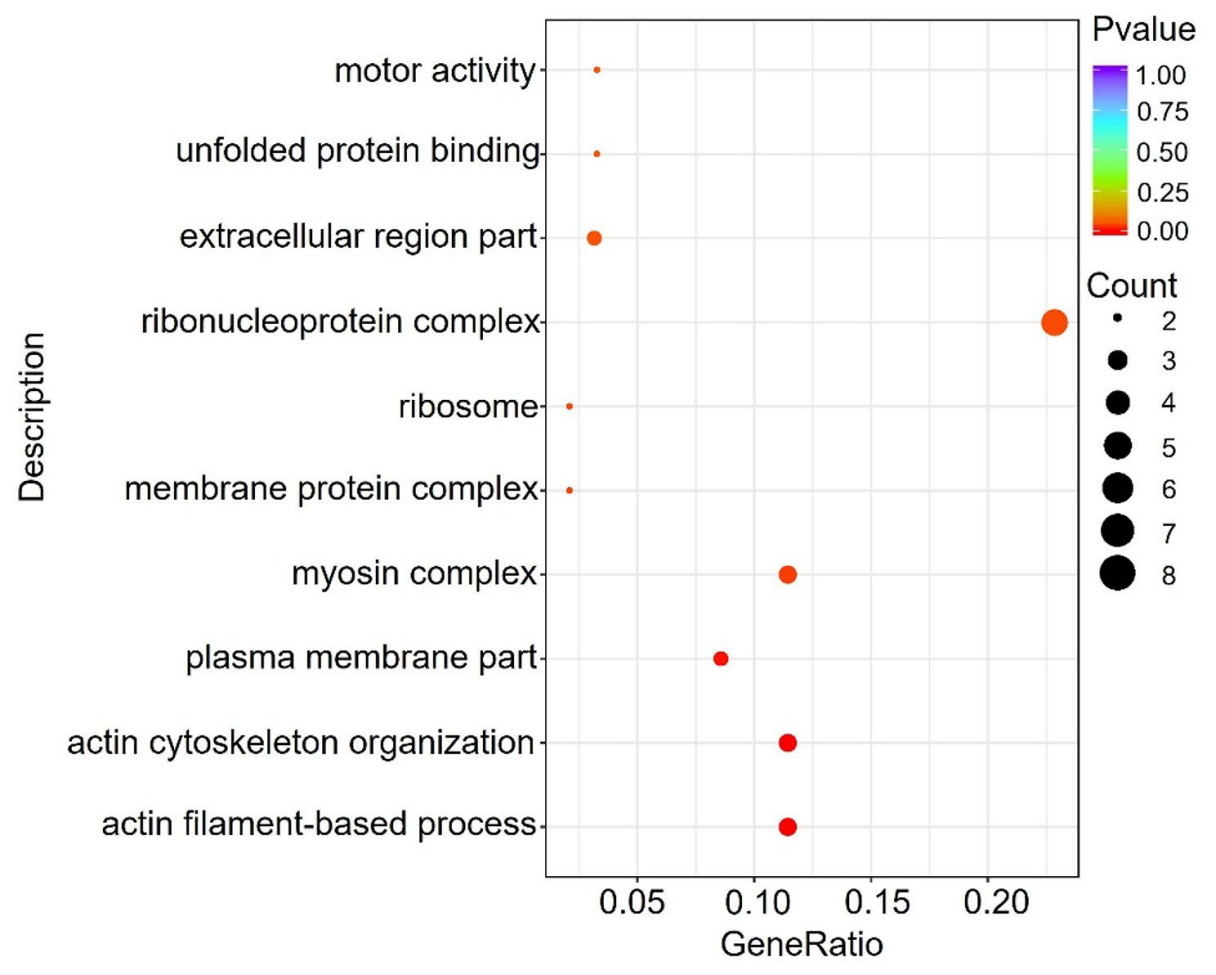
Scatterplot of GO enrichment for co-expressed differential genes

GO functional enrichment analysis and KEGG pathway enrichment analysis were performed on 191 differentially expressed genes co-expressed in the three strains. GO terms showed that DEGs were mainly involved in cell differentiation, motor activity, actin cytoskeleton organization, unfolded protein and other categories that control muscle growth and development (Fig. 3). KEGG pathway analysis showed that DEGs were significantly enriched in the FoxO signaling pathway, glycolysis / glycogen generation and glutathione signaling pathways that regulate muscle energy metabolism (Table 2). Combined with the results of GO enrichment and KEGG pathway analysis and previous studies, it is speculated that age factors may affect the growth and development of sheep through these genes and pathways.

**Table 2:** KEGG pathways common for H, SH, and EHH

KEGGID	Description	*P*-value	Count
oas04062	Chemokine signaling pathway	0.03032	5
oas00480	Glutathione metabolism	0.032269	3
oas04068	FoxO signaling pathway	0.045927	4
oas00010	Glycolysis / Gluconeogenesis	0.046856	3

### GSEA analysis

**Fig. 4:**
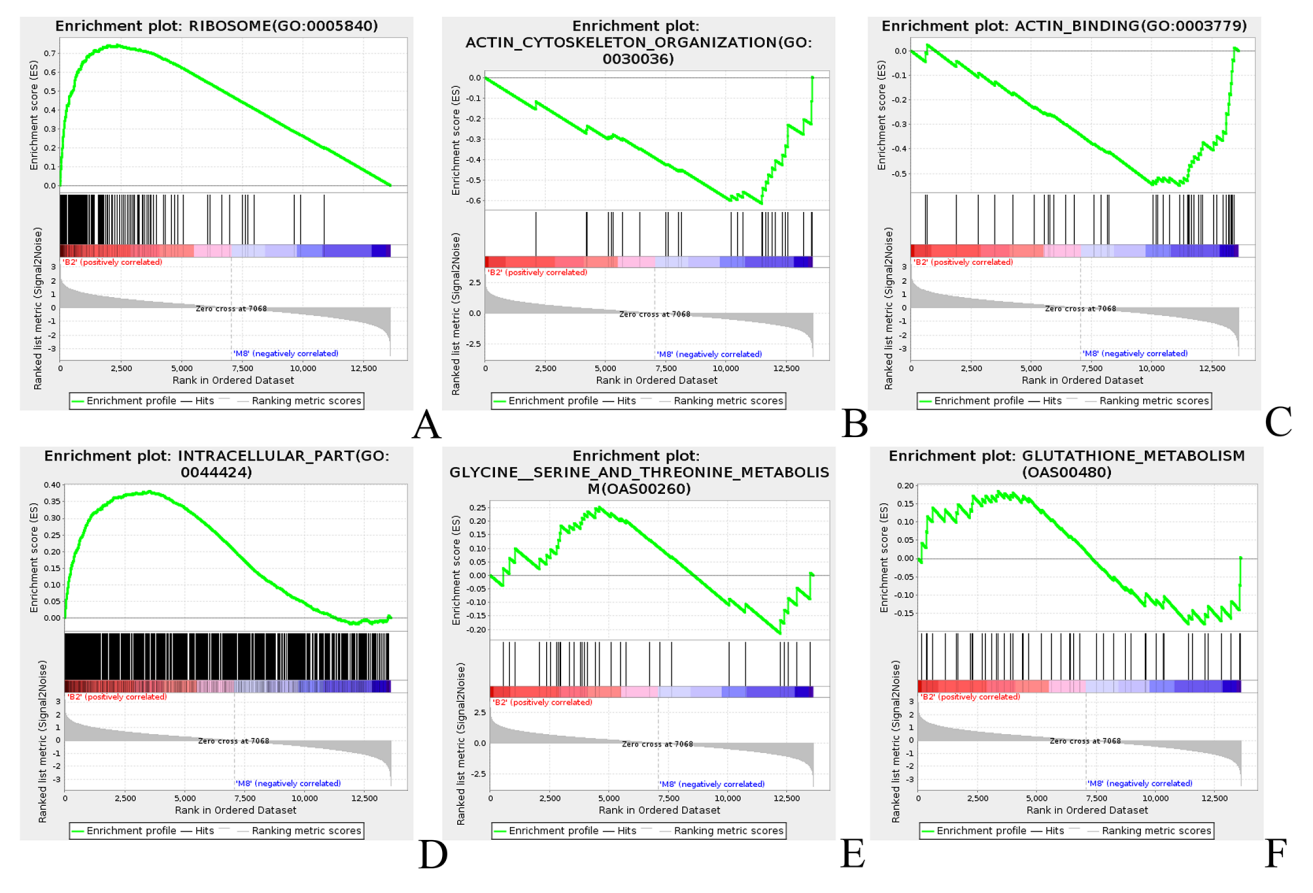
Gene Set Enrichment Analysis. A. Ribosome (GO_0005840) B. Action cytoskeleton organization (GO_0030036) C. Action binding (GO_0003779) D. Intracellular part (GO_0044424) E. Glycine serine and threonine metabolism (OAS00260) F. Glutathione metabolism (OAS00480)

GO functional enrichment pathway and KEGG enrichment pathway were analyzed by GSEA method for a total of 13,620 genes at 2 days and 8 months of age. Among them, the pathways related to muscle protein synthesis mainly involved in ribosome (Fig. 4A), actin cytoskeleton organization (Fig. 4B), actin binding (Fig. 4C), and intracellular part (Fig. 4D). The main pathways associated with skeletal muscle energy metabolism include glycine, serine, and threonine metabolism (Fig. 4E), glutathione metabolism (Fig. 4F), and glycolysis/glycogenesis signaling pathways. Compared with 8-month-old sheep, the expression levels of *DNAJA4*, *PFDN6*, *MYH8*, *CHAC1* and other genes were significantly up-regulated, while *DIAPH3*, *BPGM* and other genes were significantly down-regulated. The involvement of *DNAJA4*, *PFDN6*, *MYH8*, *CHAC1*, *DIAPH3*, and *BPGM* genes in the regulation of muscle development in sheep was further confirmed.

### Real-time fluorescence quantitative PCR proves sequencing data reliable

**Fig. 5:**
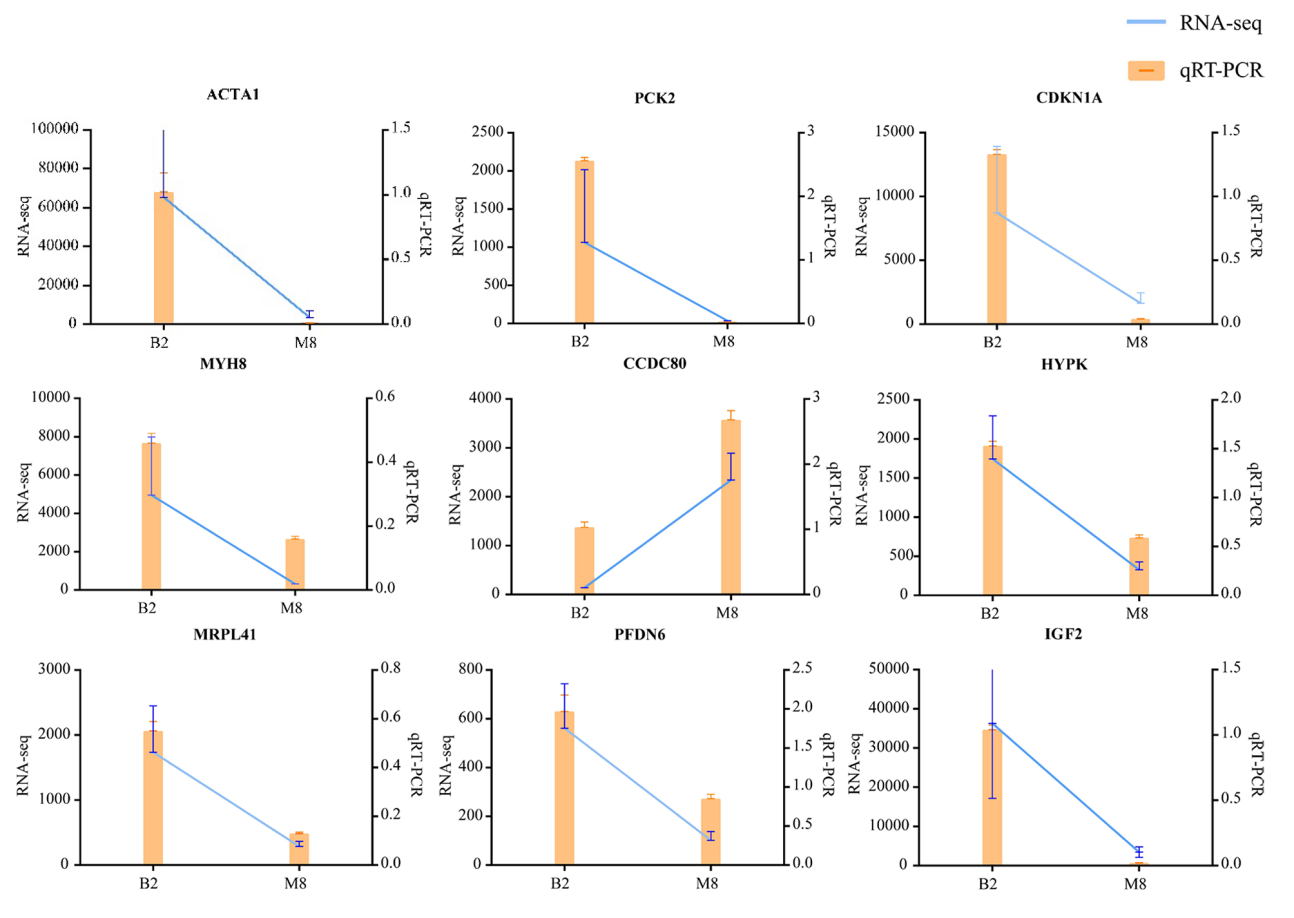
Comparison of RT-qPCR and RNA-Seq results of differentially expressed genes

To verify the reliability of the sequencing data, qRT-PCR was used to verify the randomly selected 9 highly expressed differential genes. The qPCR data were found to be consistent with the expression trends from the sequencing data in this study (Fig. 5).

## Discussion

Differences in gene expression are thought to be the one of the main reasons for affecting muscle development in animals. When gene expression changes, the regulatory mechanism of muscle development may change accordingly [11]. The aim of this study was to investigate the key genes and molecular mechanisms that regulate different growth and developmental stages of skeletal muscle. The analysis showed that 842, 1301 and 1137 differentially expressed genes were screened in the longissimus dorsi muscle of the H, SH and EHH groups at 2 days and 8 months of age, respectively. Among them, 191 genes were differentially expressed at different ages of the three sheep strains, including some genes known to be important regulators of muscle development, such as *PFDN6*, *DNAJA4* (DnaJ heat shock protein family (Hsp40) member A4), *DIAPH3* and *MYH8*.

GO functional enrichment analysis of the co-expressed genes showed that the DEGs were mainly enriched in the categories of ribosomes, motor activity, actin cytoskeleton organization, and unfolded proteins, which control muscle growth and development. The unfolded protein response (UPR) mechanism is an integrative signaling pathway with roles in promoting skeletal muscle differentiation, improving protein folding efficiency and triggering apoptosis [19, 20]. Bower NI found that *DNAJA4* and *CHAC1* (ChaC glutathione specific gamma-glutamylcyclotransferase 1) genes are involved in the trophic regulation of rapid skeletal muscle growth in Atlantic salmon, and that their expression correlates with activation of the UPR pathway [21]. Our findings show that *DNAJA4* and *PFDN6* were significantly enriched in the ribosomal signaling pathway and were up-regulated for expression in 2-day-old sheep at birth. *MYH8* encodes embryonic and neonatal myosin heavy chain, involved in skeletal muscle contraction [22]. The gene is highly expressed in the embryonic and fetal stages, and then downregulated [13], which is consistent with our bioinformatics analysis. *DIAPH3* plays an important role in remodeling the cytoskeleton and regulating cell division [23]. In proliferating cells, *DIAPH3* is required for the formation of contraction rings and clefts to achieve cell division [24–26]. The deletion of this gene will significantly affect the expression and distribution of the protein [23]. *DIAPH3* was up-regulated in 8-month-old sheep, indicating a rapid growth phase of protein translation and synthesis during this period. Our results reveal that the 2-day-old period is the prime stage of skeletal muscle protein synthesis in sheep, *DIAPH3* plays an important role in skeletal muscle growth and development of 8-month-old sheep.

The KEGG enrichment analysis of co-expressed differential genes showed that the DEGs in the three strains were significantly enriched in the FoxO signaling pathway, glycolysis / glycogen production, histidine metabolism, and glutathione signaling pathways that regulate muscle energy metabolism. Muscle development depends on changes in muscle fiber volume and number, and muscle protein accumulation is related to energy balance [27, 28]. FoxO signaling pathway is one of the main ways to regulate muscle protein degradation. Vezzali R et al. (2016) revealed that FoxO1 can activate the transcription of *CDKN1A* [29]. *CDKN1A* is involved in cell cycle regulation by interacting with proteins and is related to muscle fiber composition [30, 31]. This study found that the expression of *CDKN1A* was significantly increased at 2 days of age, suggesting that *CDKN1A* could promote muscle fiber transformation. After birth, muscle development is mainly dependent on muscle fiber hypertrophy, which is limited by inadequate oxygen supply [9]. Glycolysis plays essential role in stabilizing ATP synthesis and provides energy for muscle contraction and exercise [32]. *ALDH2* was found to be a core gene for glycolysis/glycogenesis and fatty acid degradation, which reduces apoptosis by inhibiting oxidative stress [16]. *BPGM* acts through the process of glycolysis and is an essential gene for muscle growth [33, 34]. Bioinformatics analysis showed that *ALDH2* and *BPGM* were up-regulated in 8-month-old sheep, indicating enhanced glucose utilization efficiency in 8-month-old sheep. Glutathione is a common antioxidant that has beneficial effects on maintaining muscle function [35]. It has been reported that oxidative stress can damage muscle contraction function but can increase muscle mass by increasing fiber branches [36]. The results of this study showed that *CHAC1* was enriched in the glutathione signaling pathway. *CHAC1* is a pro-apoptotic gene involved in the UPR and is significantly expressed in skeletal muscle [37]. Apoptosis and autophagy have been reported to be critical for organelle remodeling and myotube formation in the early stages of muscle differentiation [38, 39]. Li J have shown that inhibition of *CHAC1* can maintain glutathione content in skeletal muscle cells [40]. Our bioinformatics results showed that *CHAC1* was up-regulated at the 2-day-old of birth and its expression was significantly down-regulated at 8 months, indicating that *CHAC1* promotes muscle development by promoting myotube formation in the early stages of life. With age, *CHAC1* maintains muscle function by oxidation reduction. The above results showed that the DEGs mainly regulate muscle development by participating in energy metabolism at the 8-month-old stage of the three strains of sheep.

## Conclusion

In this study, RNA-Seq technology was used to sequence and analyze the transcriptome of muscle tissues of three sheep strains at 2 days and 8 months of age. GO and KEGG enrichment results showed that the differentially expressed genes were significantly enriched in muscle cell proliferation and differentiation, muscle protein synthesis and energy metabolism, indicating that the genes were mainly involved in skeletal muscle development at different developmental stages by regulating the above pathways. In this study, we investigated the effects of different developmental stages of sheep on muscle gene expression at the gene level, which enriched the studies related to muscle development and provided a basis for analyzing the mechanism of muscle development in sheep.

### Electronic supplementary material

Below is the link to the electronic supplementary material.

Supplementary Material 1

## Data Availability

The transcriptome data were deposited at the GenBank SRA database with accession number PRJNA1066495.

## Abbreviations

H: Pure breeding Hu sheep
SH: Suffolk sheep and Hu sheep hybrid F1 generation
EHH: East Friesian and Hu sheep hybrid sheep
qRT-PCR: Realtime fluorescence quantitative PCR
PFDN6: pre-folding protein subunit 6
DNAJA4: DnaJ heat shock protein family member A4
MYH8: myosin heavy chain 8
DEGs: Differentially expressed genes
FPKM: Fragments Per Kilobase of exon model per Million mapped fragments
GO: Gene Ontology
KEGG: Kyoto Encylopaedia of Genes and Genomes
DIAPH3: diaphanous related formin
CDKN1A: cyclin dependent kinase inhibitor 1 A
ALDH2: aldehyde dehydrogenase 2 family member
BPGM: bisphosphoglycerate mutase
